# When the electrocardiogram goes quiet: transient low voltage in systemic capillary leak syndrome-associated myocardial oedema—a case report

**DOI:** 10.1093/ehjcr/ytag694

**Published:** 2026-09-15

**Authors:** Peter Kordis, Vanja Persic, Andreja Cerne Cercek, Milica Lukic, Misa Fister

**Affiliations:** Clinical Department of Intensive Internal Medicine, University Medical Center Ljubljana, 1000 Ljubljana, Slovenia; Medical Faculty, University of Ljubljana, 1000 Ljubljana, Slovenia; Medical Faculty, University of Ljubljana, 1000 Ljubljana, Slovenia; Clinical Department of Nephrology, University Medical Center Ljubljana, 1000 Ljubljana, Slovenia; Clinical Department of Cardiology, University Medical Center Ljubljana, 1000 Ljubljana, Slovenia; Clinical Department of Intensive Internal Medicine, University Medical Center Ljubljana, 1000 Ljubljana, Slovenia; Clinical Department of Intensive Internal Medicine, University Medical Center Ljubljana, 1000 Ljubljana, Slovenia; Medical Faculty, University of Ljubljana, 1000 Ljubljana, Slovenia

**Keywords:** Acute heart failure, Electrocardiogram, Cardiac assist devices, Hemodynamic, Case report

## Abstract

**Background:**

Systemic capillary leak syndrome (SCLS) is a rare disorder characterized by acute episodes of hypotension, haemoconcentration, and hypoalbuminemia.

**Case summary:**

A 50-year-old woman presented with shock triggered by influenza B infection. Despite aggressive fluid resuscitation, she developed multiorgan failure and was diagnosed with severe SCLS requiring venoarterial extracorporeal membrane oxygenation due to myocardial oedema. Electrocardiography demonstrated diffuse low QRS voltage, while echocardiography revealed severe biventricular dysfunction with marked myocardial oedema. The patient was treated with immunoglobulins, corticosteroids, and plasmapheresis. Following clinical recovery, QRS voltages normalized, and cardiac function fully recovered.

**Discussion:**

SCLS is an immune-mediated rare cause of distributive shock. Cardiac involvement is uncommon but associated with high mortality. This case suggested that reversible low QRS voltage may serve as an electrocardiographic clue to myocardial oedema and severe cardiac involvement.

Learning points

- Severe systemic capillary leak syndrome may cause profound but reversible biventricular dysfunction through diffuse interstitial myocardial oedema.
- New diffuse QRS voltage attenuation may provide an early clue to severe myocardial involvement.
- Serial electrocardiogram changes should be interpreted together with echocardiography, biomarkers, and the clinical course.

## Introduction

Systemic capillary leak syndrome (SCLS) is a potentially fatal disorder characterized by episodes of hypotension, haemoconcentration, and hypoalbuminemia caused by transient systemic vascular hyperpermeability.^1^ Acute attacks are often triggered by viral infections and may lead to distributive shock, multiorgan failure, and severe oedema.^2^ Myocardial involvement can present as cardiogenic shock, likely through reversible interstitial myocardial oedema.^3^ We report a case of influenza B-triggered SCLS complicated by profound but reversible biventricular dysfunction requiring venoarterial extracorporeal membrane oxygenation (VA-ECMO). Serial electrocardiograms (ECGs) and echocardiographic examinations documented the parallel development and resolution of diffuse QRS voltage attenuation, marked myocardial thickening, and severe ventricular dysfunction.

## Summary figure

**Figure:**
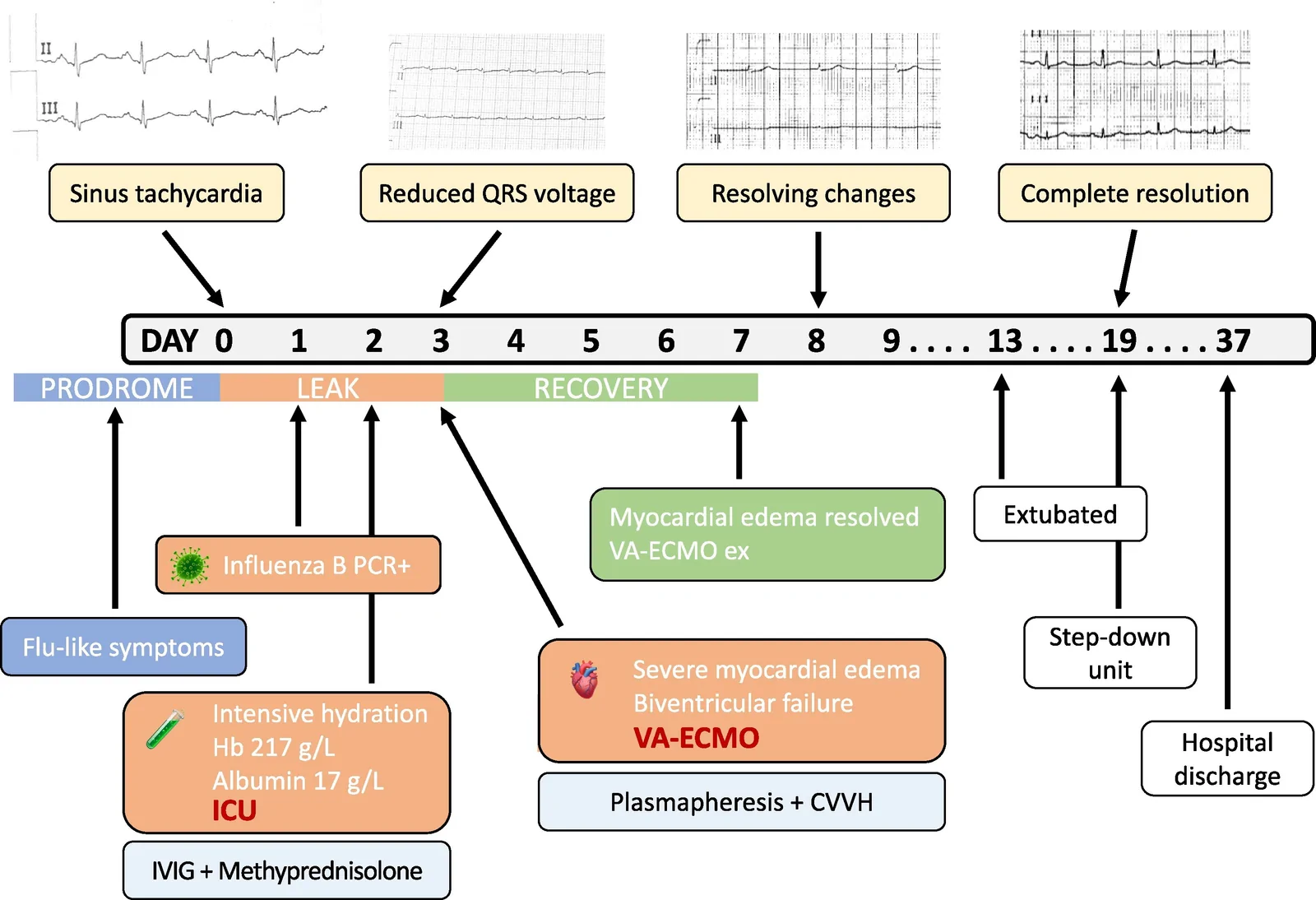


## Case presentation

**Figure 1:**
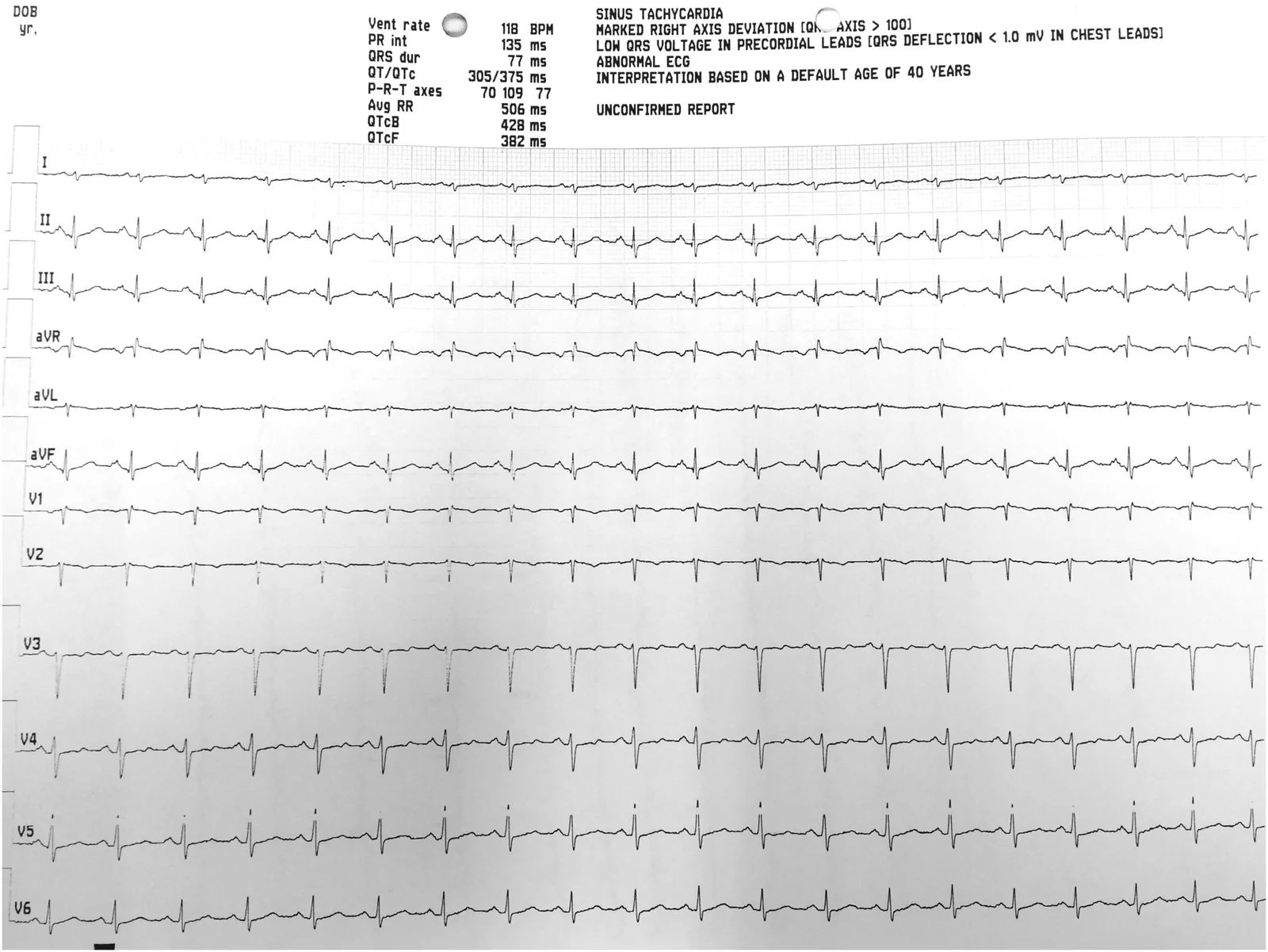
Electrocardiogram obtained at hospital admission, before the development of severe myocardial oedema, showing sinus tachycardia with preserved QRS amplitudes.

A 50-year-old woman presented to the emergency department with a 2-day history of fever up to 104° F, mild non-productive cough, sore throat, and syncope. On presentation, she was drowsy and tachycardic (heart rate 113 beats/min) with a blood pressure of 100/80 mmHg. Laboratory investigations revealed haemoconcentration (haemoglobin 170 g/L, normal 120–150 g/L), mild renal impairment, and low inflammatory markers (*Table 1*). Admission ECG showed sinus tachycardia (*Figure 1*). Her medical history was notable for asthma, treated with inhaled fluticasone and salbutamol.

**Table 1:** Laboratory values during the first 6 days of hospitalization, presented the most deranged variable in that 24-hour period

	Day 0	Day 1	Day 2	Day 3	Day 4	Day 5	Day 6
**Haemoglobin** **(120–150 g/L)**	183	214	115	118	116	115	111
Haematocrit **(0.360–0.460)**	0.503	0.653	0.337	0.355	0.337	0.336	0.340
**Platelets** **(150–410 × 10^9^/L)**	161	109	16	82	64	93	105
**Albumin** **(32–55 g/L)**		18	14	25	23	27	27
**pH** **(7.35–7.45)**		7.34	7.27	7.43	7.44	7.36	7.35
**Creatinine** **(44–97 µmol/L)**	90	88	142	185	199	193	138
**Myoglobin** **(<33.8 µg/L)**		2643	4635	9285	93.881	196.610	63.730
**CK** **(>2.41 µcat/L)**		39.9	18.7	27.0	247.0	1408.0	973.2
**Lactate** **(0.5–2.2 mmol/L)**		7.4	13.9	15.5	9.0	4.6	2.1
**Troponin T** **(<0.1 µg/L)**	0.055	1.004	3.641	10.814	8.486	13.559	8.354
**D dimer** **(<500 µg/L)**	1281	1859					
**CRP** **(0–5 mg/L)**	19	28	15	25	45	88	95
**PCT** **(0–0.5 µg/L)**	0.26	0.55	0.78	1.16	1.30	1.79	1.33
**NT-pro BNP** **(<125 ng/L)**		806					

Reference values in brackets.

CK, creatine kinase; CRP, C-reactive protein; NT-pro BNP, N-terminal pro-B-type natriuretic peptide; PCT, procalcitonin.

Chest X-ray was negative for pneumonia; however, nasopharyngeal polymerase chain reaction (PCR) was positive for influenza B, and oseltamivir was started. Despite almost 10 L of parenteral hydration administered on the ward, lactate rose to 7.4 mmol/L (normal 0.5–2.2 mmol/L), haemoglobin increased to 217 g/L, and bedside echocardiography showed persistent signs of hypovolemia with hyperdynamic circulation. Albumin was 18 g/L (normal 32–55 g/L) and inflammatory markers remained low, including serum interleukin-6 levels.

**Figure 2:**
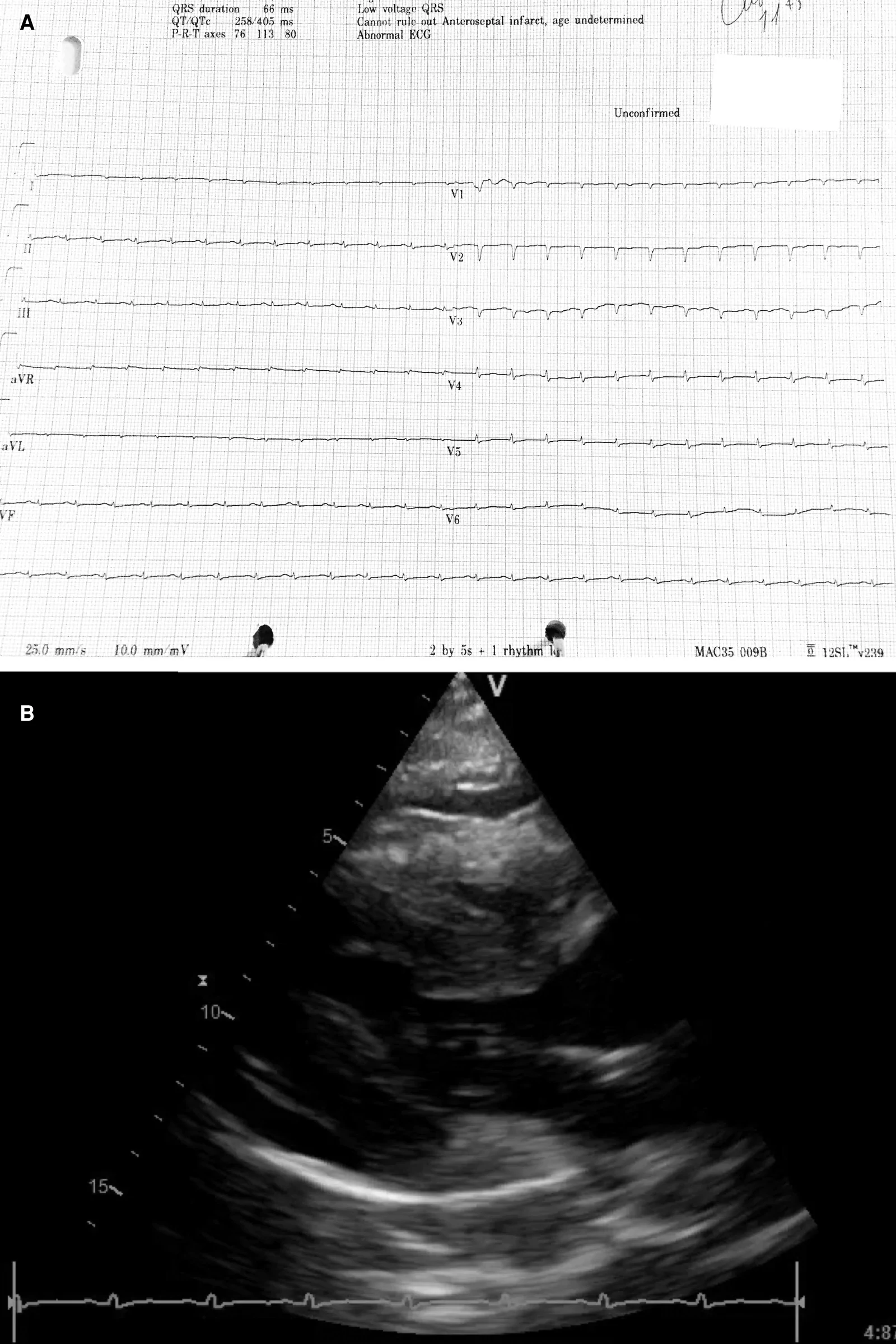
(*A*) Electrocardiogram obtained on day 3 before venoarterial extracorporeal membrane oxygenation initiation. Compared with the admission tracing, there is marked diffuse QRS amplitude attenuation with subtle repolarization abnormalities. (*B*) Transthoracic echocardiography on the same day during mixed shock. Parasternal long-axis view showing marked diffuse myocardial thickening consistent with oedema, with left ventricular wall thickness of up to 20 mm, severe global biventricular systolic dysfunction, and moderate pericardial effusion.

The patient was transferred to the intensive care unit for suspected SCLS. Parenteral hydration and albumin substitution were continued, but extensive fluid administration led to generalized oedema, most pronounced in the extremities, without definite signs of compartment syndrome. Intravenous immunoglobulins (IVIG) (0.4 g/kg) and methylprednisolone (0.8 mg/kg) were initiated after review of the literature. The patient subsequently required intubation and urgent haemodialysis for metabolic acidosis. Although haemoglobin levels decreased, shock persisted despite escalating vasopressor doses. Electrocardiogram revealed diffusely reduced QRS voltage (*Figure 2*). Follow-up echocardiography showed severe biventricular failure with diffuse myocardial oedema, left ventricular wall thickness of up to 2 cm, normal chamber sizes, left ventricular ejection fraction (LVEF) of 10%, and left ventricular outflow tract velocity time integral (LVOT VTI) of 3 cm. A moderate pericardial effusion was present without echocardiographic signs of tamponade (*Figure 2*). The patient was started on dobutamine, but required venoarterial extracorporeal membrane oxygenation (VA-ECMO) support on day 3 because of worsening cardiogenic and distributive shock and rising serum lactate levels. Coronary angiography was normal, and troponin I remained modest relative to the severity of ventricular dysfunction (*Table 1*).

**Figure 3:**
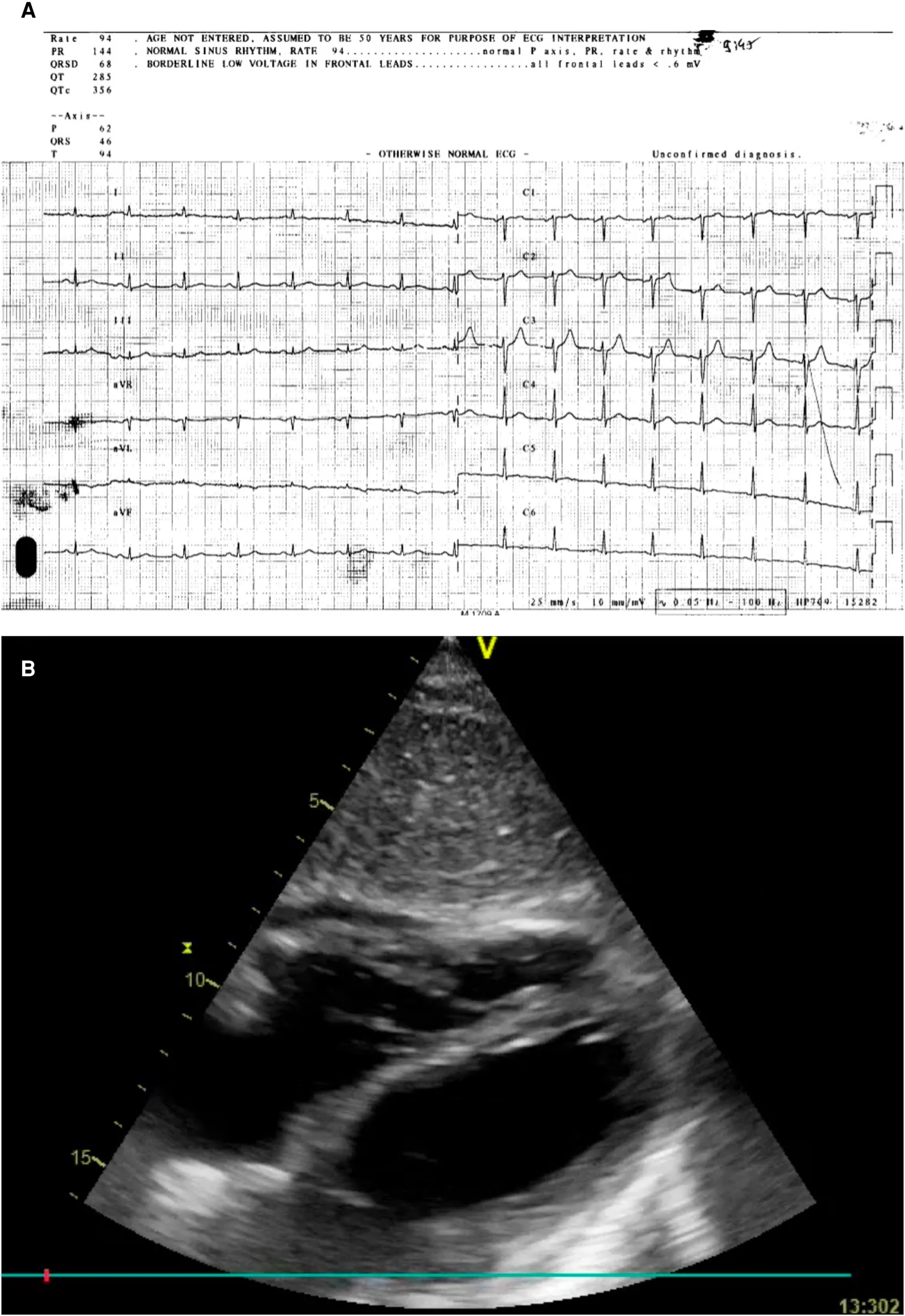
(*A*) Follow-up electrocardiogram obtained after clinical and echocardiographic recovery, showing restoration of QRS amplitudes. (*B*) Follow-up echocardiography (substernal view) on day 8 showed substantial regression of myocardial oedema and recovery of ventricular systolic function.

Methylprednisolone and IVIG were continued for 5 days, and two plasmaphereses were performed. Continuous venovenous hemofiltration (CVVH) was initiated to achieve a negative fluid balance. In the following days, myocardial oedema regressed, with improvement in global left ventricular ejection fraction (*Figure 3*). ECG changes resolved in parallel, with normalization of QRS voltage (*Figure 3*). The patient was weaned off ECMO support on day 7 and extubated on day 13. The ICU stay was complicated by *Serratia marcescens* ventilator-associated pneumonia and probable pulmonary aspergillosis. On day 19, the patient was transferred to a step-down unit and discharged to a rehabilitation centre on day 37. At discharge, renal function was normal, and LVEF was estimated at 60%.

In 2025, the patient experienced a milder episode of SCLS following influenza A infection. She had previously been vaccinated, and oseltamivir was started early. Although she was admitted after an episode of syncope, she did not require vasoactive or respiratory support. Echocardiography showed preserved left ventricular ejection fraction without overt myocardial wall thickening. Cardiac magnetic resonance imaging (MRI) performed on day 1 of hospitalization demonstrated preserved biventricular systolic function, diffusely elevated native T1 and T2 values consistent with myocardial oedema, and subtle segmental subepicardial late gadolinium enhancement (LGE) without convincing evidence of myocardial necrosis. There was no pericardial thickening or effusion. Her clinical condition and myocardial oedema resolved after 5 days of treatment with IVIG and methylprednisolone. No QRS voltage changes were observed during the second flare.

## Discussion

This case demonstrates typical red-flag features of severe SCLS, defined by hypotension, haemoconcentration, and hypoalbuminemia in the absence of other identifiable causes of shock.^1^ The pathogenesis is poorly understood but may be associated with a trigger releasing pro-inflammatory cytokines that promote vascular permeability.^4^ Immunomodulatory treatment with IVIG, methylprednisolone, and plasmapheresis was based on published reports of severe SCLS.^2,5,6^

**Figure 4:**
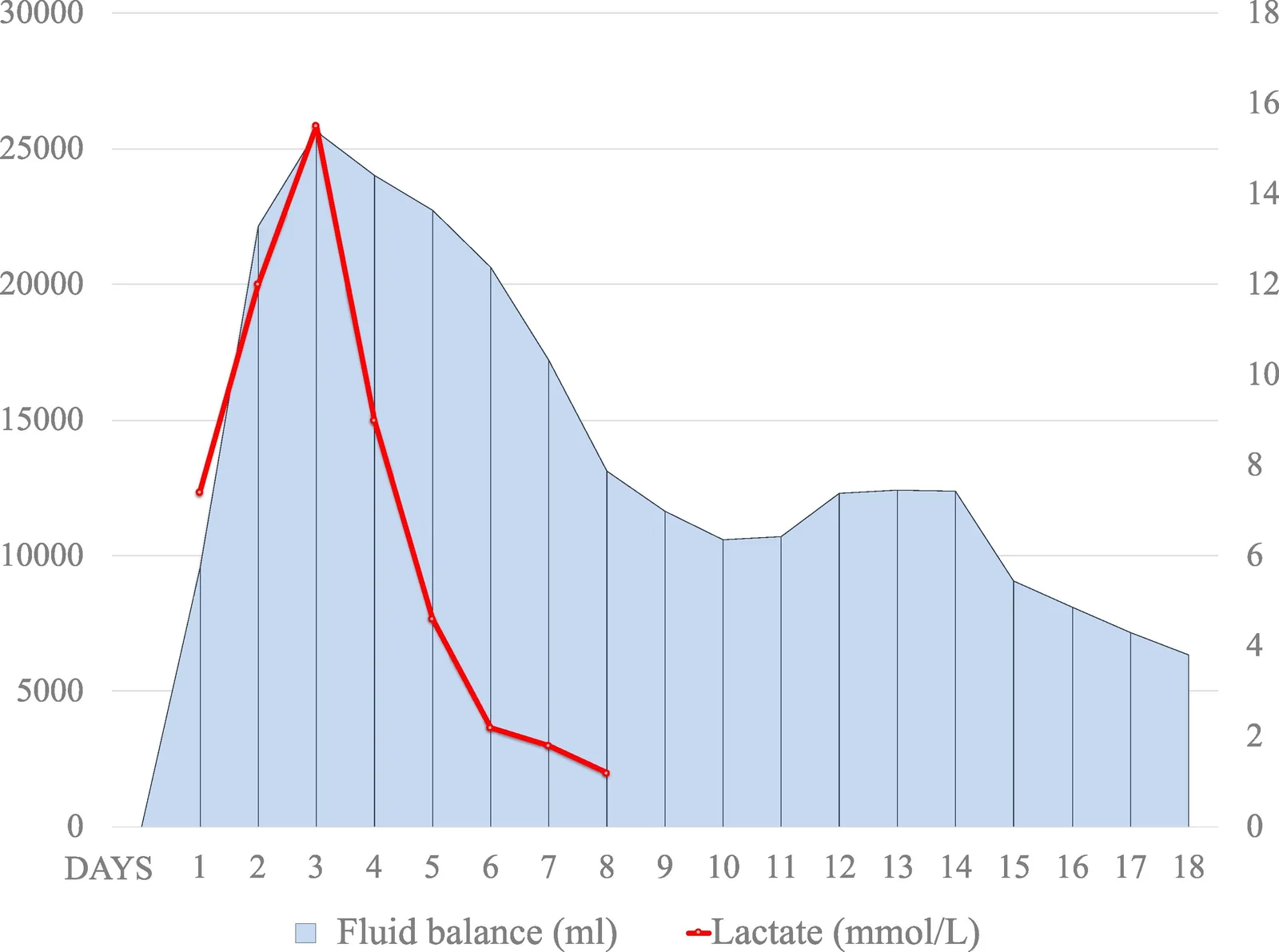
Cumulative fluid balance and serum lactate concentration during the first 18 days of hospitalization. Marked positive fluid balance accumulated during the extravasation phase because of persistent intravascular depletion. Lactate peaked during refractory shock and declined following venoarterial extracorporeal membrane oxygenation initiation, immunomodulatory treatment, and fluid removal.

The syndrome usually has three phases—prodrome, extravasation, and recovery. The most common trigger appears to be an upper respiratory tract infection in up to half of reported cases.^2,5,6^ Extravasation is the most threatening phase, causing distributive shock and severe systemic oedema, with a median duration of 3.8 days.^2^ Our patient received over 25 L of crystalloids and colloids within the first 3 days due to intravascular hypovolemia (*Figure 4*). She was anuric throughout the extravasation phase. In the largest ICU-treated cohort, cumulative fluid administration over 10.7 L was associated with higher mortality.^3^ Pulmonary oedema, compartment syndrome, and rhabdomyolysis are the major causes of mortality.^7^ In our patient, severe oedema and threatening compartment syndrome prompted early CVVH; haemodynamics improved during fluid removal and recovery.

Severe episodes of SCLS may be complicated by transient myocardial dysfunction. In a report of 37 patients with flares requiring ICU treatment, cardiogenic shock was described in four cases, one of which resulted in cardiac arrest.^3^ In 2018, the European Clarkson Registry reported myocardial dysfunction in 22% of severe cases. The median left ventricular ejection fraction was 15%, 50% had pericardial effusion, and transient hypertrophy was reported in three cases. Six of the 14 patients required circulatory support (intra-aortic balloon pump, ECMO, or Impella), and four patients died. Follow-up echocardiography was normal in all survivors.^8^ The exact pathogenesis of myocardial involvement is not fully understood, but several cases have reported myocardial oedema. In one case, myocardial biopsy during the first reported use of central ECMO in a patient with cardiogenic shock demonstrated diffuse interstitial oedema without inflammatory infiltrates or necrosis.^9^

The differential diagnosis of ventricular dysfunction in our patient included influenza-associated fulminant myocarditis, septic cardiomyopathy, and Takotsubo syndrome. Influenza myocarditis was considered plausible because the infection preceded cardiac dysfunction. However, echocardiography initially showed hyperdynamic biventricular function with profound hypovolemia, indicating distributive shock, while ventricular dysfunction developed later and was disproportionate to the initial biomarker elevation. Cardiac MRI during the second SCLS episode showed diffuse myocardial oedema with only subtle subepicardial LGE and no convincing evidence of necrosis; the recurrent pattern therefore favoured capillary-leak-associated myocardial involvement over prior fulminant myocarditis. Septic cardiomyopathy and Takotsubo syndrome were considered less likely because inflammatory markers remained low with no bacterial source identified, while ventricular dysfunction was global and biventricular without a characteristic ballooning pattern and was accompanied by marked myocardial oedema. Endomyocardial biopsy was considered to exclude fulminant myocarditis but was not performed because of severe haemodynamic instability and thrombocytopenia. As the result would not have changed immediate management, the procedural risk was considered to outweigh the potential benefit. Consequently, concomitant inflammatory myocardial involvement cannot be definitively excluded.

Electrocardiographic findings in severe SCLS have rarely been reported and have not been systematically characterized. Only sporadic case reports have described low QRS voltage in association with severe myocardial dysfunction and myocardial oedema.^9,10^ Juthier *et al*. documented low QRS voltage and reversible biventricular dysfunction in a patient with confirmed myocardial oedema.^9^ More recently, Nobata *et al*. reported COVID-19-associated SCLS with diffuse low QRS voltage and echocardiographic myocardial oedema that resolved with clinical recovery.^10^ In our patient, serial ECGs documented normal QRS voltage before deterioration, extremely low QRS amplitudes and ST-segment abnormalities during the acute phase, and normalization after recovery. The concomitant pericardial effusion showed no signs of tamponade and was unlikely to explain the degree of voltage reduction. During the second, milder SCLS flare, myocardial oedema occurred without QRS attenuation, suggesting that voltage reduction may reflect the severity of myocardial involvement. Serial ECG assessment may therefore provide a simple bedside clue to severe cardiac involvement in SCLS.

In-hospital mortality of severe SCLS episodes ranges from 19% to 23%, while 5-year survival is 73–76%.^3,7,8^ Despite prolonged hospitalization, our patient had a favourable outcome, with minimal residual sensory deficits in the lower extremities.

Diffuse low QRS voltage is non-specific and may result from various conditions. However, in patients with suspected SCLS and haemodynamic compromise, a rapid reduction in QRS amplitude may indicate worsening myocardial oedema and prompt urgent echocardiographic reassessment. Low voltage should therefore be regarded as a warning sign rather than a stand-alone diagnostic criterion. In our patient, QRS attenuation paralleled myocardial oedema and biventricular failure, while voltage normalization coincided with recovery. This case highlights the potential value of serial ECGs in monitoring severe SCLS with myocardial involvement.

## Lead author biography


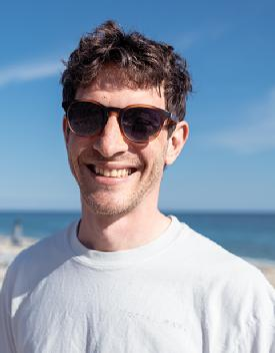
Peter Kordis graduated from the Faculty of Medicine, University of Ljubljana, and completed specialization in cardiology and intensive care medicine. He obtained a postgraduate degree focusing on antiplatelet treatment after cardiac arrest. His research interests include post-cardiac arrest care, mainly neuroprognostication. He currently works at the University Medical Center Ljubljana.

## Data Availability

The data underlying this article are available in the article. No additional datasets were generated or analysed.
